# Molecular regulatory mechanisms and diagnostic potential of dendritic cell-derived exosomes in liver transplantation: from immune tolerance induction to translational challenges

**DOI:** 10.3389/fimmu.2025.1657956

**Published:** 2025-08-28

**Authors:** Xiaopeng Chen, Zhiqi Yang, Minghao Li

**Affiliations:** ^1^ Department of Hepatobiliary Surgery, People's Hospital of Ningxia Hui Autonomous Region, Ningxia Medical University, Yinchuan, Ningxia, China; ^2^ Third Clinical Medical College, Ningxia Medical University, Yinchuan, Ningxia, China

**Keywords:** dendritic cells, exosomes, liver transplantation, immune tolerance, clinical translation

## Abstract

Liver transplantation remains the only curative treatment for end-stage liver disease (ESLD); however, immune rejection significantly hampers its long-term success. Dendritic cell-derived exosomes (DEXs) have emerged as a promising tool for inducing immune tolerance and enabling precise immunomodulation in liver transplantation, owing to their unique bidirectional immunoregulatory capabilities. This review systematically summarizes the biological characteristics and functional properties of DEXs, with a particular focus on their multidimensional regulatory mechanisms within the hepatic transplant immune microenvironment. These include: the mechanisms and pathways by which DEXs mediate immune tolerance; the synergistic immunoregulatory roles of DEXs and exosomes derived from other immune cells. Furthermore, we explore the potential of DEXs for integrated diagnostic and therapeutic applications, engineering upgrades to treatment strategies, and their prospects for clinical translation. Despite their promise, several challenges persist, including difficulties in exosome isolation and purification, prolonged preparation times, bioengineering limitations, and the lack of effective *in vivo* tracking methods. We propose that advancements in artificial intelligence, biomaterials science, and interdisciplinary technologies may help overcome these barriers, facilitating the precise isolation, functional optimization, and clinical translation of DEXs. This review emphasizes the molecular immunoregulatory networks governed by DEXs and discusses their translational pathways, aiming to promote individualized diagnostic and therapeutic strategies in liver transplantation.

## 1 Introduction

Liver disease causes more than 2 million deaths worldwide each year, accounting for approximately 4% (1 in 25) of all deaths globally (1). Both chronic injury (viral hepatitis, alcoholic liver disease) and acute injury (acetaminophen overdose) can lead to progressive deterioration of the liver’s structure and function, with the end stage known as ESLD (2). Currently, there are no approved drugs that can reverse or halt the progression of ESLD (3). Therefore, liver transplantation remains the only curative option (4). Recent advances in surgical techniques and perioperative management have dramatically improved the short-term success rate of liver transplantation.However, its long-term efficacy continues to be hindered by the imbalance between immune rejection and immune tolerance (5). The ideal immune state following liver transplantation is the establishment of immune tolerance, which allows the transplanted liver to function stably without continuous immunosuppressive therapy (6). In clinical practice,however, only about 20%–40% of liver transplant recipients achieve so-called “operational tolerance.” (7) Although the widespread use of immunosuppressive drugs has significantly reduced the incidence of acute rejection, their long-term administration is associated with serious side effects such as infections, tumorigenesis,and metabolic disorders, which severely affect patients’ quality of life (8). Therefore, the development of safer and more precise immunomodulatory strategies has become a core direction in liver transplantation research.

As an “immune-privileged organ”, the liver possesses a unique immune microenvironment that can promote graft-specific immune tolerance by modulating the function of local immune cells, such as dendritic cells (DCs) (9). DCs are recognized as the most potent antigen-presenting cells (APCs), capable of capturing, processing, and presenting antigens to naive T cells, thereby activating helper T cells or cytotoxic T cells to initiate antigen-specific immune responses (10). Serving as a critical bridge between innate and adaptive immunity, DCs not only enhance T cell-mediated rejection through antigen presentation (11) but also promote the differentiation of regulatory T cells (Tregs) and contribute to immune homeostasis (12). The immunological function of DCs—either promoting immune activation or inducing immune tolerance—largely depends on their maturation status. *In vitro*-generated exosomes from mature DCs typically activate T cells and enhance immune responses, whereas those from immature DCs tend to induce T-cell anergy or promote immune tolerance (13). In this review, the terms “immature” and “mature” DCs refer to bone marrow-derived or monocyte-derived DCs generated *in vitro*. *In vivo*, these correspond to steady-state or activated DCs, respectively. *In vivo* studies in humans have shown that donor-derived regulatory DCs(DCregs) may modulate host APCs, memory CD8^+^ T cells, and Tregs, potentially contributing to immune regulation at the time of transplantation (14). More recently, DCs have been found to secrete exosomes, which retain several biological properties of their parent cells (15) and carry surface molecules such as major histocompatibility complexes (MHCs), miRNAs, and various immunomodulatory proteins (16). These DEXs exhibit immunoregulatory functions comparable to those of DCs themselves. Depending on their cellular origin, DEXs may exert bidirectional immunomodulatory effects: exosomes derived from mature DCs promote antigen-specific T-cell activation and proliferation, thereby facilitating graft rejection (17, 18), whereas exosomes derived from immature DCs suppress anti-donor immune responses, prolong graft survival, and demonstrate a tolerogenic potential (19). This bidirectional regulatory capacity positions DEXs as key modulators in maintaining immune homeostasis in the context of transplantation.

As an emerging platform for integrated diagnostics and therapeutics, exosomes exhibit several unique biological advantages. Their phospholipid bilayer structure provides effective protection against enzymatic degradation; surface markers enable tissue-specific chemotaxis and targeted delivery; and their luminal contents—such as miRNAs and lncRNAs—serve as noninvasive biomarkers reflecting the immune status of both donors and recipients (20–22). Notably, exosomal miRNA profiles in the plasma of liver transplant recipients have been found to correlate closely with the severity of graft rejection (23). In addition to their diagnostic utility, exosomes can function as nanoscale delivery platforms for therapeutic agents, including small molecules and gene-editing tools. Their ability to traverse biological barriers and deliver cargo precisely to immune targets offers a promising strategy for achieving “invisible” immune modulation (24, 25). In conclusion, DEXs not only hold great promise in promoting immune tolerance following transplantation, but also represent a versatile theranostic platform—simultaneously serving as biomarkers and targeted delivery vehicles—to advance precision immunomodulation in liver transplantation.

## 2 Biological properties and functions of DEXs

In several Murine transplantation models, “cross-dressed” recipient APCs—bearing intact donor major histocompatibility complex(MHC) molecules transferred via small extracellular vesicles released from allogeneic grafts—have been described as a key mechanism in initiating and sustaining allogeneic immune responses (26). Experimental evidence suggests that this mechanism may underlie the spontaneous acceptance of liver allografts in the absence of immunosuppressive therapy (27).

DEXs are enriched with immunoreactive components, including miRNAs, antigen-presenting molecules(MHC-I and MHC-II), and co-stimulatory molecules (CD80/CD86), which enable them to directly or indirectly modulate immune responses (28). The molecular composition and immunological functions of DEXs are highly dependent on the maturation state of their parental DCs. Exosomes derived from *in vitro*-generated immature DCs exhibit pronounced immunosuppressive properties. For instance, in a murine kidney transplantation model, Exosomes derived from *in vitro*-generated immature DCs enriched with miR-682—a microRNA that negatively regulates Rho-associated coiled-coil containing protein kinase 2 (ROCK2)—promote the differentiation of Tregs and suppress pro-inflammatory cytokines such as IL-2, IL-17, and IFN-γ, ultimately prolonging graft survival (29). Moreover, exosomes derived from *in vitro*-generated immature DCs contain various immunosuppressive proteins, including milk fat globule epidermal growth factor VIII (MFGE8), which mitigates systemic pro-inflammatory responses by enhancing phagocytosis and secondary immunosuppression (30). Conversely, exosomes derived from *in vitro*-generated mature DCs demonstrate a more potent immune-activating capacity. These exosomes carry pre-loaded major histocompatibility complex class I (MHC-I) and class II (MHC-II) molecules, as well as co-stimulatory molecules (CD80/CD86), which can be transferred to recipient cells. While they do not actively process or present antigens, the transferred peptide–MHC complexes can modulate antigen presentation by recipient APCs (31). Via MHC-I and MHC-II molecules, exosomes derived from *in vitro*-generated mature DCs deliver antigenic signals to naive CD8^+^ cytotoxic T lymphocytes(CTLs) and CD4^+^ helper T cells(Th), respectively, thereby inducing antigen-specific immune responses (32, 33). Additionally, exosomes derived from*in vitro*-generated immature DCs carry immune-activating miRNAs such as miR-155, which further potentiate their immunostimulatory effects (34).

**Figure 1:**
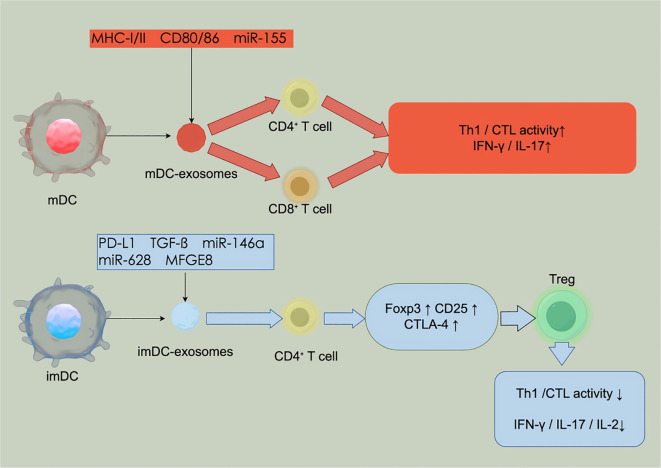
Dual immunoregulatory roles of DEXs in liver transplantation.

This molecular compositional plasticity arises from DC-specific cytosolic sorting mechanisms that selectively enrich distinct miRNAs and functional proteins, enabling precise immunomodulation tailored to specific microenvironmental cues (32). Functionally, DEXs not only display MHC molecules on their surface but also express programmed death ligand 1(PD-L1) (12). This allows them to present donor antigens and activate both CD4^+^ and CD8^+^ T cells, inducing rejection responses (35, 36), while also inhibiting T cell activation through PD-L1–CD80 trans interactions, exerting a negative regulatory effect (37). Together, this DEX-centered immunoregulatory network plays a pivotal role in maintaining immune homeostasis and regulating graft tolerance. In summary, the highly tunable molecular features and functional diversity of DEXs provide a solid molecular foundation and theoretical framework for the development of individualized immunoregulatory strategies in liver transplantation through refined immune modulation mechanisms (**Figure 1**). In addition, DEXs possess the unique capacity to both activate immune responses and regulate immune tolerance. In one study, administration of interleukin-10(IL-10)-induced DEXs significantly suppressed the development of collagen-induced arthritis, mitigating inflammatory responses and tissue damage in a murine model (38). These findings suggest that exosomes derived from exosomes derived from immature DCs can effectively inhibit inflammatory and autoimmune responses.

Conversely, DEXs sensitized with Toxoplasma gondii antigens were shown to specifically localize to the spleen following adoptive transfer in mice, where they elicited a robust Th1-type antigen-specific immune response that markedly enhanced host resistance to infection (39). Furthermore, another study demonstrated that DEXs loaded with tumor-associated antigenic peptides successfully activated antigen-specific CTLs, which subsequently eradicated or suppressed tumor growth via a T cell-dependent mechanism (40).

Collectively, these results highlight the dual immunomodulatory capabilities of DEXs in the context of liver transplantation: not only can they facilitate the induction of immune tolerance to prevent graft rejection, but they can also potentiate protective immune responses against infections and malignancies when appropriately stimulated. This functional versatility positions DEXs as promising tools for both immune suppression and immune activation in transplant immunotherapy.

## 3 Mechanisms of DEXs involved in the immune microenvironment of liver transplantation

In a rat liver transplantation model exosomes from donor-derived steady-state DCs have been shown to induce immune tolerance, which is further enhanced when combined with Treg cells (41). DEXs act as critical regulators within the complex immune microenvironment of liver transplantation, dynamically modulating immune responses through multidimensional mechanisms to maintain the delicate balance between immune tolerance and rejection.

### 3.1 Mechanisms and pathways for the establishment of DEXs-mediated immune tolerance

Small extracellular vesicles released shortly after liver transplantation have been shown to exert immunosuppressive effects, whereas those released at later stages lack such activity, potentially influencing the immunogenicity of liver grafts (42). Donor-derived DEXs can enter the recipient’s circulation post-transplantation and migrate to secondary lymphoid organs, including the spleen and lymph nodes. The donor antigenic peptide-MHC complexes and immune co-stimulatory or inhibitory molecules (PD-L1) expressed on their surfaces directly interact with recipient T cells to modulate their activation or inhibition (43). For example, exosomes derived from immature or tolerant DCs generated *in vitro* may be enriched in immunosuppressive factors such as TGF-β1 and CTLA-4, which inhibit T cell activation and proliferation and modulate other immune cells(recipient DCs), synergistically promoting Treg differentiation and maintaining immune tolerance (44, 45), notably, immature dendritic cells can exhibit either tolerogenic or immunogenic properties, depending on the specific stimuli and the surrounding microenvironment. It was shown that donor-derived DEX increased the proportion of Tregs and the expression of Foxp3 mRNA in recipient spleens in a mouse kidney transplantation model (29). Furthermore, PD-L1 carried by exosomes can bind to PD-1 on T cells, suppressing their proliferation and cytotoxicity (46). This cell-free antigen delivery mechanism bypasses the need for direct cell contact, providing unique advantages over traditional pathways. Transforming growth factor β1(TGF-β1) contained within exosomes effectively induces naive T cell differentiation into CD25^+^CTLA-4^+^Foxp3^+^ Tregs, thereby exerting immunosuppressive effects (47). Mechanistically, TGF-β activates the Smad2/3 signaling pathway, upregulating peroxisome proliferator-activated receptor γ coactivator 1α(PGC-1α), which promotes mitochondrial fusion and inhibits hypoxia-inducible factor 1α(HIF-1α). This leads to increased expression of carnitine palmitoyltransferase 1(CPT1), a key enzyme in fatty acid oxidation(FAO), and decreased expression of glycolytic enzyme hexokinase 2(HK2), thus reprogramming T cell metabolism from glycolysis toward FAO (48). Such metabolic remodeling supports Treg differentiation and function, establishing a foundation for immune tolerance.

DEXs also regulate T cell subset differentiation via delivery of specific noncoding RNAs. For example, miR-125a and miR-125b target and inhibit the transcription factor STAT3, thereby suppressing Th17 cell differentiation (49), while TGF-β enrichment promotes Treg expansion by inducing Foxp3 expression (50). Additionally, DEXs modulate inflammatory responses by targeting the TRAF6/IRAK-1/NLRP3 pathway, reducing secretion of pro-inflammatory cytokines such as IL-1β (51). In a murine colitis model, DEX miR-146a regulates Treg function through the IFN-γ/STAT1 signaling pathway, attenuating Th1 immune responses (52, 53). This non-cell-contact-dependent, targetable modulation mediated by exosomal noncoding RNAs holds great potential for gene-level immunointervention.

Moreover, DEXs facilitate the polarization of macrophages from a pro-inflammatory M1 phenotype to an immunosuppressive M2 phenotype, thereby enhancing the local immunosuppressive microenvironment (50). This indicates that DEXs exert immunoregulatory effects not only on T cells but also by remodeling the broader immune milieu, providing a molecular basis for tolerance maintenance. Collectively, DEXs play a central immunomodulatory role in establishing immune tolerance after liver transplantation through multiple mechanisms, including antigen presentation, inhibitory signaling, metabolic reprogramming, noncoding RNA regulation, and immune microenvironment remodeling.

However, it should be noted that most evidence supporting DEX-mediated tolerance induction arises from rodent models. Human liver transplant recipients often present with underlying conditions such as viral hepatitis or fatty liver disease, generating chronic inflammatory microenvironments potentially impairing exosomal immunomodulatory function. For example, HBeAg-positive chronic hepatitis B patients exhibit significantly higher Treg levels than HBeAg-negative patients (54), suggesting that species differences and disease heterogeneity may influence therapeutic efficacy.

### 3.2 Synergistic immunomodulation by DC-derived and multicellular exosomes in liver transplantation

Although DEXs have received considerable attention in the context of liver transplantation due to their potential to induce immune tolerance, exosomes from other cellular origins also play significant roles in modulating immune cell function and may act synergistically with DEXs to shape the perigraft immune microenvironment. Studies have demonstrated that hepatocyte-derived exosomes can alleviate hepatic inflammatory responses and promote regulatory Treg expansion in murine models (55). Furthermore,THP-1 monocytes pretreated with hepatocyte-derived exosomes exhibited a marked reduction in proinflammatory cytokine expression—such as IL-8 and IL-1β—upon lipopolysaccharide(LPS) stimulation (56). These endogenous exosomes may support the differentiation of anti-inflammatory immune subsets, thereby functionally cooperating with exosomes derived from immature DCs to promote Treg induction and suppress pro-rejection responses. Additionally, tumor-derived exosomes carrying immunosuppressive factors such as prostaglandin E2(PGE2) and transforming growth factor-β(TGF-β) can be internalized by bone marrow progenitor cells, promoting the differentiation of monocytes into DCs and the expansion of myeloid-derived suppressor cells, which inhibit T cell activity (57, 58). In hepatocellular carcinoma (HCC), TEXs have been shown to reprogram macrophages via activation of the NF-κB signaling pathway, leading to increased secretion of proinflammatory cytokines and the formation of M2-polarized tumor-associated macrophages (59). Moreover, tumor-derived exosomes can reprogram neutrophils and macrophages, simultaneously suppressing IFN-γ and TNF-α production while upregulating PD-1 expression on T cells, thus contributing to an immunosuppressive phenotype (60). Although the immunological mechanisms of tumor-derived exosomes are primarily associated with tumor immune evasion, their ability to modulate the immune microenvironment suggests potential relevance in the context of transplantation tolerance. These findings imply that DEXs and exosomes from diverse cellular sources may collaboratively modulate immune responses in the liver transplant microenvironment through synergistic regulatory pathways.

## 4 A new paradigm for immunotherapy and diagnostics in liver transplantation

DEXs exhibit significant theranostic potential in the context of liver transplantation, owing to their unique capabilities in immune regulation, biomarker-based diagnosis, and targeted therapeutic delivery. These properties position them as a transformative platform in the development of next-generation strategies for transplantation immunotherapy.

### 4.1 Diagnostic potential and marker validation

DEXs have been extensively investigated as biovectors for non-invasive biomarker screening in liquid biopsies, owing to their high accessibility, structural stability, and resistance to enzymatic degradation. Exosomes are widely distributed in blood and other body fluids, and their phospholipid bilayer membranes protect encapsulated proteins and microRNAs (miRNAs) from degradation by extracellular nucleases and proteases, thus ensuring their integrity and diagnostic utility. In the field of organ transplantation, a critical clinical challenge is the lack of real-time and accurate methods to assess graft immune status. Donor-derived exosomes, particularly those originating from DCs, have been proposed as promising biomarkers for early immune responses following transplantation—including acute and chronic rejection. Their potential lies in enabling early detection and dynamic monitoring of immune rejection events, facilitating timely clinical intervention and potentially improving graft survival (61). In-depth analysis of DEXs derived from *in vitro* models (BMDCs/MoDCs) can reveal molecular signatures—such as miRNA and proteomic profiles—associated with distinct DC activation states(steady-state vs. activated). These signatures could serve as reference maps to interpret compositional changes in sEVs/DEXs directly isolated from patient biofluids (blood, bile), thereby enabling inference of the *in vivo* activation status of DCs and the prevailing immune microenvironment, and supporting the non-invasive diagnosis of graft rejection or tolerance. Recent studies have demonstrated that the miRNA profiles carried by DEXs closely reflect the post-transplant immune status. For example, bone marrow-derived dendritic cell exosomes are enriched in miR-34a and miR-21 (62), the latter of which is significantly upregulated during episodes of rejection and shows a strong positive correlation with immune activity (63). Moreover, downregulation or inhibition of miR-21 has been shown to exert anti-inflammatory effects by activating the STAT3 signaling pathway, promoting macrophage polarization toward the immunosuppressive M2 phenotype (64). Compared to exosomes from *in vitro*-generated immature DCs, those from *in vitro*-generated mature DCs are enriched in immunoregulatory miRNAs such as miR-146a, miR-125b-5p, and miR-148a-3p (65–67). Notably, miR-148a-3p has been suggested as a potential diagnostic biomarker for chronic graft-versus-host disease (68).In a murine transplant model, miR-146a was shown to Treg function through the IFN-γ/STAT1 pathway, with miR-146a deficiency associated with prolonged graft survival and reduced rejection severity (52). Additionally, miR-155 is significantly upregulated in exosomes from human monocyte-derived mature DCs compared to those from imDCs (69). Both miR-155 and miR-146a are strongly implicated in graft immune responses and have been identified as non-invasive biomarkers for chronic graft-versus-host disease, with high expression levels observed during active disease states (70). These findings indicate that DEX miRNA expression profiles can not only provide dynamic, time-resolved monitoring of immune status but also differentiate between distinct immunological conditions—such as acute rejection, chronic tolerance, and graft-versus-host disease—thereby offering precise molecular insights and potential diagnostic markers for immune surveillance in liver transplantation.

However, it is important to note that exosomal miRNA signatures can be influenced by fluctuations in hepatic function. For instance, patients with cirrhosis often exhibit elevated baseline levels of plasma exosomal miR-21 (71), which may obscure rejection-associated expression changes and increase the risk of false-negative interpretations. Therefore, the establishment of standardized baseline correction models will be essential for improving the accuracy and clinical applicability of exosome-based diagnostics in the future. At present, the exploration of DEXs/sEVs as diagnostic markers in liver transplantation remains at an early and largely speculative stage, requiring validation through extensive clinical studies.

### 4.2 Engineering upgrades to treatment strategies

DEXs not only exhibit passive cargo delivery capabilities but can also be genetically engineered to achieve enhanced functionality and targeted immunoregulation (38, 72–74). Accumulating evidence suggests that genetically modified DCs and their exosomal products can significantly suppress graft rejection and promote immune tolerance following transplantation. For instance, CD80^+^ DEXs have been shown to attenuate acute rejection after liver transplantation by downregulating the expression of the NLRP3 inflammasome and suppressing CD8^+^ T cell adhesion, infiltration, and proinflammatory cytokine production (75). In another study, a lentiviral vector system was employed to overexpress miR-193b-3p in DCs. The resulting exosomes were enriched with this miRNA and were capable of significantly increasing the proportion of Tregs, while simultaneously suppressing NLRP3 expression, decreasing the levels of proinflammatory cytokines such as IL-1β and IL-17A, and upregulating anti-inflammatory cytokines including IL-10 and TGF-β. These changes effectively mitigated immune rejection in a liver transplantation model (76).

Collectively, these findings demonstrate the potential of engineered DEXs as programmable, cell-free delivery vehicles for immunoregulatory molecules. Their ability to be customized at the molecular level highlights a promising platform for precise and durable immunomodulation in liver transplantation and beyond. DCs express CCR7, which guides their homing to lymph nodes via CCL19/CCL21 signaling, thereby enabling preferential interactions with T cells and other APCs in secondary lymphoid organs (77, 78). Enhancing CCR7 expression has been shown to markedly increase the accumulation of DEXs in secondary lymphoid organs and strengthen their interactions with T cells. For instance, in a myocardial infarction model, CCR7-high DEXs(MI-DEXs) exhibited greater splenic accumulation and an enhanced capacity to activate CD4^+^ T cells, inducing IL-4 and IL-10 expression as demonstrated by near-infrared imaging (79). Another study on dendritic cell vaccines demonstrated that nano-exosomes(Hy-M-Exo) fused with cell membranes expressing CCR7 exhibited significantly greater targeted accumulation in lymph nodes *in vivo* compared with conventional exosomes, and increased uptake by APCs(including DCs) in lymph nodes by approximately 1.7-fold (80). This evidence supports the mechanism by which the CCR7–CCL19/CCL21 axis mediates the targeting of DEXs to lymphoid organs and facilitates their interactions with T cells or APCs, providing a basis for the optimization of targeting strategies.

### 4.3 Translational prospects in clinical practice

In recent years, DEXs have emerged as a promising vector for developing novel immune tolerance-inducing strategies, owing to their low immunogenicity, high biological stability, and potential for engineered modifications. In various preclinical models related to liver transplantation, DEXs have demonstrated favorable bioactivity and safety profiles, underscoring their high translational potential.

For instance, in a murine model of liver ischemia–reperfusion (IR) injury, systemic administration of DEXs via tail vein successfully delivered heat shock protein 70 (HSP70) to naive T cells, activated the PI3K/mTOR signaling pathway, and regulated the Treg/Th17 cell balance. This intervention significantly attenuated IR-induced hepatic injury and preserved liver structure and function, providing a promising strategy to mitigate IR injury during transplantation (81).

Moreover, in a rat orthotopic liver transplantation model, the combined infusion of donor-derived exosomes derived from immature DCs and donor antigen–specific Tregs induced and sustained long-term transplantation tolerance without the use of conventional immunosuppressive agents (38). This approach not only circumvented the adverse effects and infection risks associated with long-term immunosuppressive therapy but also highlighted the unique capacity of DEXs to induce antigen-specific immune tolerance *in vivo*.

## 5 Current challenges and future directions

The successful clinical translation of current DEX therapies necessitates overcoming several critical bottlenecks. These include the standardization of exosome isolation protocols, reduction of production timelines, development of reliable long-term *in vivo* tracking technologies, and improvement of drug-loading stability.

For instance, the preparation cycle for DCreg-based therapy at the University of Pittsburgh requires approximately three weeks (14), which significantly exceeds the typical decision-making window for cadaveric donor liver transplantation—usually less than 72 hours (82). (**Table 1**) Summarizes the core discrepancies between the current technological capabilities and the clinical demands, underscoring the urgent need for innovative approaches to accelerate and optimize DEX manufacturing and application workflows.

**Table 1:** Key technical bottlenecks and clinical requirements for clinical translation of DEXs.

Methodological step	Existing approach	Clinical demand	Gap
High separation specificity	Ultracentrifugation: 30–70% recovery (83); Microfluidics: >80%, but may retain polymer residues (84).	GMP-compliant standardized production (≥95% purity, contamination-free)	Functional validation of contaminant effects
*In Vivo* Tracking	Relies on techniques such as ICG labeling (duration: ~72 h) (85)	Real-time monitoring (≥30 days) (23)	The lack of long-term, multi-target tracking capabilities.
Preparation Time	21 days for rat exosomes derived from immature DCs model preparation (38)	Donor liver transplantation protocols demand rapid processing (<72 h) (82)	A significant mismatch with urgent clinical requirements.
Drug Loading Stability	Ultrasound-assisted loading can compromise membrane integrity (86)	High-efficiency drug loading ;minimal cellular damage	Process complexity

### 5.1 technological bottleneck

Current isolation techniques for DEXs continue to face significant technical bottlenecks and methodological controversies. Due to their nanoscale size, low density, and fragile membrane structures, achieving efficient isolation and purification while maintaining biological activity remains a critical challenge in the field. At present, the predominant separation strategies include differential ultracentrifugation (DUCC), size-exclusion chromatography (SEC), and immunoaffinity capture (IAC), among these, DUCC has long been regarded as the “gold standard” due to its ability to preserve exosomal membrane integrity. However, its limitations—such as prolonged operation time, low throughput, and suboptimal purity—pose significant obstacles to meeting the demands of clinical standardization and large-scale production (87, 88). The SEC method, which separates particles based on size, offers advantages such as operational simplicity and minimal sample perturbation. Nevertheless, when target vesicles approach or exceed the upper limit of the chromatographic medium’s pore size, the resolution diminishes significantly, thereby compromising exosome purity (83). Immunoaffinity capture, while highly specific, suffers from issues related to the heterogeneous expression of surface antigens across exosomal subpopulations and the potential for incomplete removal of bound antibodies, which can interfere with downstream functional analyses (89). In recent years, viscoelastic microfluidics has emerged as a promising non-labeled, continuous-flow, and high-throughput alternative. This method employs biocompatible polymers added to the suspension medium to generate size-dependent elastic lift forces that enable the selective enrichment of exosomes, achieving >90% purity and recovery rates exceeding 80% (84). However, residual polymers may contaminate biological samples and negatively impact the structural and functional validation of isolated exosomes. It should be noted that the reported “enrichment” of specific molecules(miRNAs, proteins) in DEXs in many studies is typically based on comparisons with their parental DCs or with the total composition of the culture supernatant. However, the biological relevance and specificity of such “enrichment” should be interpreted with caution, as strictly matched, unpurified vesicle populations (total sEVs or conditioned medium) are often lacking as controls. Ideally, future studies should incorporate more comprehensive and appropriately matched comparison groups.

For *in vivo* tracking applications, the FDA-approved near-infrared fluorescent dye indocyanine green(ICG) has been successfully utilized for exosome labeling. For instance, HGI@Exo formulations enable real-time imaging of exosome biodistribution in liver transplant recipients (85). While this approach enhances visualization of homing and biodistribution, further refinement is required to minimize potential alterations in the exosomes intrinsic biological functions due to the labeling process.

To date, most exosome-tracking methods rely heavily on fluorescence-based techniques, which are limited by issues such as channel number constraints, spectral overlap, low signal flux, and poor multiplexing capabilities (90). Thus, the development of more sensitive, stable, and biologically compatible imaging technologies remains an urgent priority for advancing the clinical translation of exosome-based therapeutics.

### 5.2 Engineering dilemma

Exosomes have demonstrated remarkable potential as drug delivery vehicles; however, their engineering remains encumbered by several technical and translational challenges (91). Currently, widely adopted drug-loading strategies—such as ultrasound-assisted methods—are considered relatively mild and have been shown to enhance drug encapsulation efficiency by transiently remodeling the exosomal membrane without significantly disrupting protein or lipid components (92). Nonetheless, prolonged or excessive ultrasound exposure may compromise the structural and functional integrity of exosomal membranes, thereby reducing their biological stability (86). Endogenous cargo loading through genetic engineering has also emerged as a promising strategy. For instance, tumor-derived exosomes have been employed to deliver CRISPR/Cas9 systems targeting poly(ADP-ribose) polymerase 1 (PARP-1), yielding potent therapeutic outcomes in solid tumor models (93). However, this approach is technically complex, costly, and difficult to scale, thereby limiting its feasibility for routine clinical application and industrial-scale production.

To address these limitations, there is a pressing need to develop more efficient, robust, and scalable exosome drug-loading technologies. Recent efforts have focused on integrating nanocomposite-modified microfluidic platforms with innovative approaches such as cellular nanoperforation, exosome-enveloped protein nanocage (EPN) capture systems, and tunable optimized particle-enhanced exosome vesicle technology. These emerging strategies aim to enable high-loading capacity, structural stability, and high-throughput engineered production of exosomes to meet clinical translational demands (94).

### 5.3 Future perspectives: toward precision and integration

With advancements in artificial intelligence(AI) and bioinformatics,exosome identification and function prediction models based on machine learning have been increasingly applied to cancer staging, disease diagnosis, and therapeutic target discovery (95). Recent studies have explored the integration of exosomal miRNA expression profiles with AI algorithms to enable early prediction of rejection in renal transplantation (96, 97). This concept holds significant potential for extension to liver transplantation, where the dynamic profiling of exosomal miRNAs, in combination with single-cell sequencing technologies and machine learning models, may facilitate the development of intelligent early warning systems for predicting postoperative immune rejection. In parallel, the rapid evolution of interdisciplinary technologies—such as cryo-electron microscopy (98), high-resolution microfluidics (99), and AI-assisted data analytics (100)—is paving the way for the construction of a comprehensive “precision isolation–functional validation–clinical translation” workflow. This approach would enable the systematic classification and targeted modulation of exosomal functional subpopulations. Moreover, combining exosomes with biomaterials—such as hydrogel-based sustained-release systems—may enhance their *in vivo* half-life and enable localized remodeling of the immune microenvironment within specific tissues (101). Additionally, optimization of freeze-drying and preservation techniques has been shown to improve the structural stability of exosomal proteins and RNAs (102), thereby supporting the feasibility of storage, transport, and formulation for large-scale, multi-center randomized controlled trials (RCTs). These advances will be essential for validating the safety and efficacy of DEXs in preventing acute rejection after liver transplantation.

## 6 Conclusion

DEXs have emerged as a promising immunotherapeutic modality in liver transplantation, owing to their unique immunomodulatory capabilities. By delivering key regulatory cargos—such as miRNAs, immunosuppressive proteins, and surface ligands—DEXs modulate both innate and adaptive immune responses, including the activity of T cells and myeloid cell subsets. This enables them to exert a dual role:inducing graft-specific immune tolerance while simultaneously enhancing host defense against infections and malignancies. In preclinical models, DEXs have demonstrated the ability to attenuate ischemia-reperfusion injury and prolong allograft survival without the need for conventional immunosuppressive agents. Furthermore, advances in bioengineering have enabled the customization of DEXs for targeted delivery and functional augmentation, underscoring their potential as both diagnostic biomarkers and therapeutic vectors. However, several translational challenges persist, including low-yield isolation, exosome heterogeneity, and limited delivery efficiency. Notably, there is a paucity of clinical studies evaluating the safety, biodistribution, and immunological efficacy of human-derived DEXs in liver transplantation. Future investigator-initiated trials(IITs) are warranted to validate preclinical findings and assess clinical applicability. Looking ahead, the integration of microfluidic technologies, advanced imaging, artificial intelligence, and biocompatible scaffolds may enable large-scale production, high-resolution functional profiling, and personalized application of DEXs. With continued technological refinement and multicenter clinical validation, DEXs are poised to become a critical component of individualized immunotherapy and immune monitoring in liver transplantation.
