# Correction: Molecular regulatory mechanisms and diagnostic potential of dendritic cell-derived exosomes in liver transplantation: from immune tolerance induction to translational challenges

**DOI:** 10.3389/fimmu.2025.1707327

**Published:** 2025-10-06

**Authors:** Xiaopeng Chen, Zhiqi Yang, Minghao Li

**Affiliations:** ^1^ Department of Hepatobiliary Surgery, People’s Hospital of Ningxia Hui Autonomous Region, Ningxia Medical University, Yinchuan, Ningxia, China; ^2^ Third Clinical Medical College, Ningxia Medical University, Yinchuan, Ningxia, China

**Keywords:** dendritic cells, exosomes, liver transplantation, immune tolerance, clinical translation

The funder “Ningxia Natural Science Foundation”, “2025AAC020056” to “Xiaopeng Chen, Zhiqi Yang and Minghao Li” was erroneously omitted.

The original version of this article has been updated.

